# Chorioretinal Side Effects of Therapeutic Ocular Irradiation: A Multimodal Imaging Approach

**DOI:** 10.3390/jcm9113496

**Published:** 2020-10-29

**Authors:** Giulia Midena, Raffaele Parrozzani, Luisa Frizziero, Edoardo Midena

**Affiliations:** 1 UOC Oftalmologia, Fondazione Policlinico Universitario A. Gemelli IRCCS, 00168 Rome, Italy; giulia.midena@gmail.com; 2 Department of Ophthalmology, University of Padova, 31044 Padova, Italy; raffaele.parrozzani@unipd.it; 3 IRCCS-Istituto di Ricovero e Cura a Carattere Scientifico—Fondazione Bietti, 00198 Rome, Italy; lfrizziero@gmail.com

**Keywords:** ocular oncology, radiation chorioretinopathy, radiation retinopathy, radiation maculopathy, radiation optic neuropathy, optical coherence tomography (OCT), optical coherence tomography angiography (OCTA)

## Abstract

Radiation chorioretinopathy, radiation maculopathy, and radiation optic neuropathy are the major complications of ophthalmic radiotherapy. Optical coherence tomography (OCT) and OCT angiography (OCTA) are revolutionary imaging methods, allowing the visualization of the retinal cellular architecture and the retinal vascular system, respectively. In recent years this multimodal imaging approach has been applied to several retinal disease, but its role in the clinical characterization of retinal complications secondary to ophthalmic radiotherapy has not yet been defined. The purpose of this review is to critically evaluate the role of OCT and OCTA in the clinical assessment of radiation-induced chorioretinopathy, maculopathy, and optic neuropathy.

## 1. Introduction

**Figure 1:**
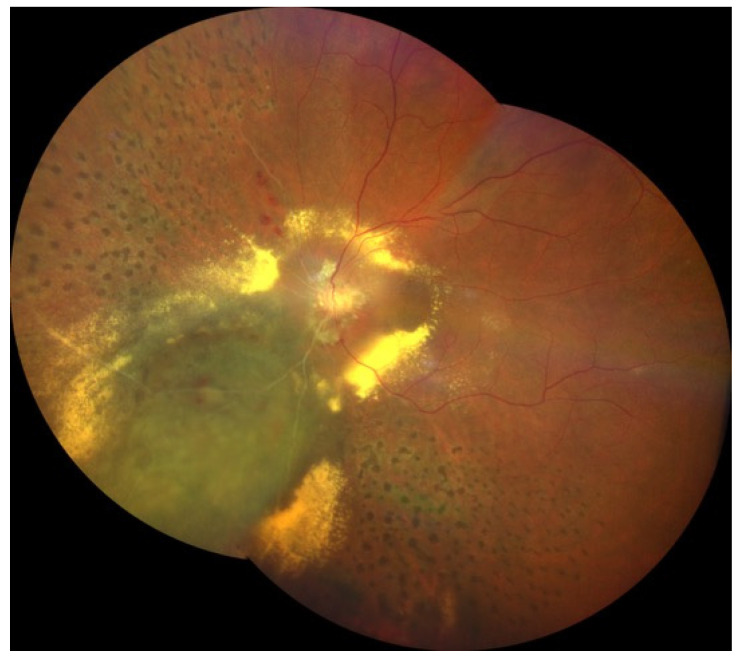
Color fundus photography showing a laser-treated radiation chorioretinopathy, characterized by chorioretinal atrophy, ischemia, ghost vessels, cotton wool spots, hard exudates and retinal hemorrhages; hemorrhages and hard exudates involve both the macular region (radiation maculopathy) and the optic nerve (radiation optic neuropathy).

**Figure 2:**
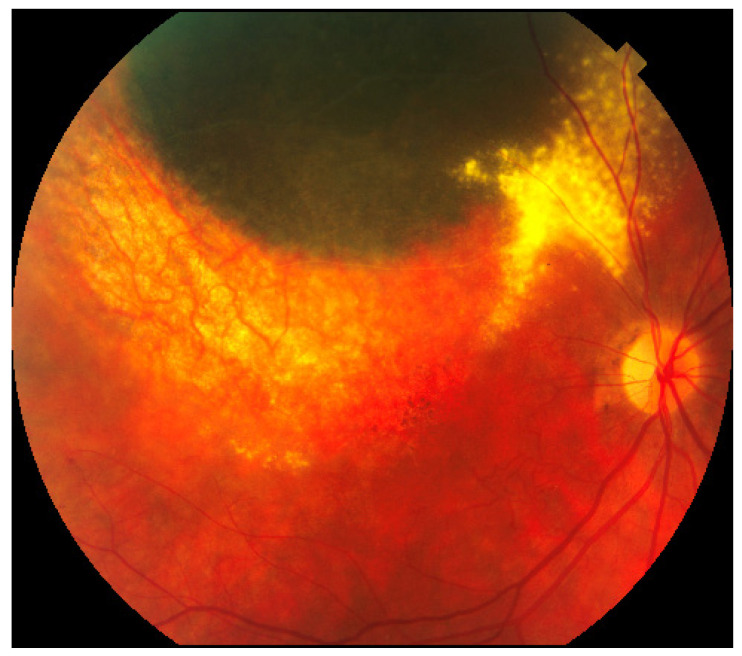
Color fundus photography showing radiation maculopathy in an advanced stage, characterized by chorioretinal and retinal pigment epithelium atrophy in the macular area.

**Figure 3:**
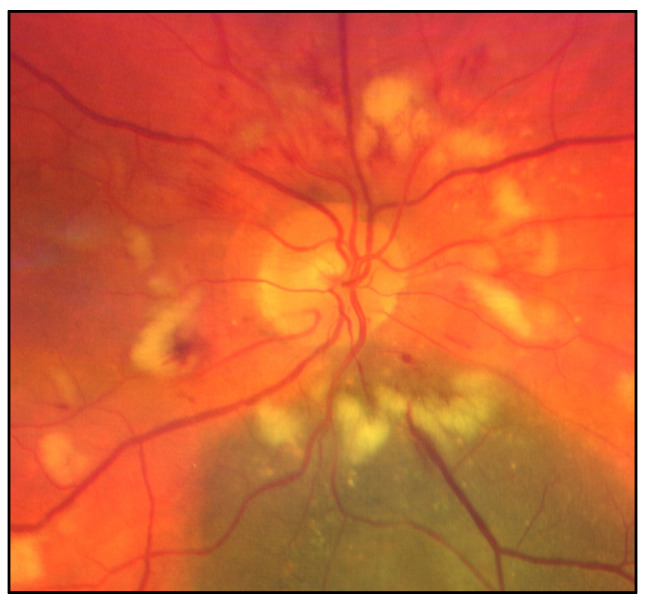
Color fundus photography showing radiation optic neuropathy, characterized by optic disc edema, peripapillary hemorrhages and peripapillary exudates. The treated uveal melanoma is visible as a pigmented lesion inferior to the optic nerve.

Imaging of the posterior segment of the eye is playing a fundamental role in ocular oncology, not only in the differential diagnosis of intraocular tumors, but also in the clinical characterization of ocular side effects related to the intraocular tumor treatment (mainly irradiation), providing valuable structural, functional and morphological information on the chorioretinal complex and the visual pathway [1]. Nowadays, radiotherapy is the standard of care for most patients affected by uveal melanoma (UM), the most common primary intraocular malignant tumor of adults [2,3]. Although radiotherapy offers an eye-sparing alternative for these patients, the Collaborative Ocular Melanoma Study (COMS) reported that 3 years post-treatment, nearly 50% of patients had a visual acuity of 20/200 or worse [2,3]. The clinical presentation of chorioretinal damage, due to eye irradiation, occurs from 6 months to 5 years after radiotherapy, and this damage may cause visual acuity loss (42% at 5 years) [4]. Notwithstanding, it has been demonstrated that chorioretinal subclinical changes occur earlier [4]. The prevention and the management of these complications are still limited, but in the retinal multimodal imaging era it may be useful to have a precocious identification and classification of major chorioretinal complications due to eye irradiation: Radiation chorioretinopathy (Figure 1), radiation maculopathy (Figure 1 and Figure 2), and radiation optic neuropathy (Figure 1 and Figure 3) [5,6,7,8,9,10].

This review was planned to report the pathophysiology of radiation damage and the main imaging tools in ocular oncology, and to revise the major chorioretinal complications due to eye irradiation (radiation chorioretinopathy, maculopathy and optic neuropathy), focusing on optical coherence tomography (OCT) and optical coherence tomography angiography (OCTA) findings.

### 1.1. Uveal Melanoma and Radiotherapy

Radiotherapy is currently the gold standard in the treatment of UM. Although enucleation may be indicated for some large tumors, most medium and small sized UM are treated with ocular irradiation. There is no consensus about patients’ counselling about the various types of irradiation [1,4,6,9], but the most common modalities are (plaque) brachytherapy and proton beam radiotherapy. Both these techniques may be used to treat the vast majority of UM [10,11,12,13,14].

Ocular irradiation causes both tumor regression and iatrogenic side effects on the surrounding healthy tissues, mainly retina and choroid. It is not yet fully understood how radiotherapy damages these tissues, but literature supports a combination of at least two mechanisms: Microvascular occlusion by direct irradiation effect and a diffuse neuroinflammation due to cytokines/chemokines secretion by the irradiated tumor cells. Although the neural retina components, like the central nervous system neural cells, are quite resistant to radiation damage, the retinal and choroidal vessels are particularly sensitive to it [9]. Therefore, the primary sites of damage secondary to radiotherapy are the endothelial cells of the retina, choroid and optic nerve, inducing a progressive vessel obliteration. This histologic aspect of microvascular occlusion is the same observed in brain irradiated capillaries, and in the typical retinal occlusive microvascular disorders, as diabetic retinopathy. Retinal blood vessels present outpouchings, fusiform teleangectasia and microaneurysms. Vascular incompetence results in vascular leakage, edema and hard exudates. Moreover, narrowing or closure of the capillary lumen causes retinal ischemia. Eventually, retinal atrophy develops. Choroidal vessels may also show beadings, telangiectatic-like dilatations, microaneurysms and vessel sclerosis [9,10,11,12,13]. Recently, it has been proposed that cellular stress due to irradiation induces DNA damage of tumor cells leading to cell “senescence”, that is the main mechanism of solid tumor regression after radiotherapy. At the same time most of the senescent cells remain active for several months and years and acquire a “senescence-associated secretory phenotype”. This phenotype promotes the interaction between senescent cells and their microenvironment, supporting a local inflammatory response that involves also healthy (vascular) cells [14].

### 1.2. Main Imaging Tools in Ocular Oncology

OCT is a non-invasive, depth-resolved technique that allows to image retinal morphology at 5 µm resolution, producing cross-sectional and three-dimensional images of the central retina [15]. This technique is now well established in the diagnosis, management and treatment of almost all retinal diseases, mainly to quantify the amount of macular edema and to visualize the retinal pigment epithelium (RPE) and neuroretina status [16]. OCT is useful to identify and grade radiation macular edema, since its early stages, and correlate it with visual acuity [17,18]. Despite conventional OCT allowing the visualization of the anatomic changes in the macular area, it offers poor contrast between static tissue and blood vessels in most retinal layers. Therefore, structural OCT is not used to identify vascular changes such as capillary rarefaction, macular ischemia or neovascularization.

In order to visualize vascular structures and their morphologic/functional changes, the most commonly used tools in clinical practice are fluorescein (FA) and indocyanine green angiography (ICGA). FA is typically employed to visualize the retinal blood vessels, while ICGA is used to study choroidal ones. FA remains the gold standard to demonstrate radiation retinopathy, mainly detecting retinal ischemia related to this disease, while both FA and ICGA are preferred when the optic nerve is irradiated [4]. Both these imaging techniques require intravenous dye injection, that can give rise to some systemic adverse side effects, and is also time consuming [19,20].

In order to develop a non-invasive, dye-free method to visualize chorioretinal vasculature, a number of extensions of OCT have been probed. OCTA represents the newest development in the field of retinal imaging. OCTA has gradually replaced FA and ICGA in the diagnosis of many retinal diseases because it is able to penetrate into the chorioretinal layers and image the different capillary plexuses. OCTA allows to reconstruct and visualize choroid and retinal vessels in a three-dimensional representation, separating the different retinal and choroidal plexuses, layer by layer [21].

These considerations suggest the importance to critically evaluate the role of OCT and OCTA in clinical ocular oncology practice, better defining their role and utility in the assessment of retinal complications due to eye irradiation.

## 2. Materials and Methods

To identify potentially relevant articles in the medical literature, we searched MEDLINE**^®^** (8600 Rocksville Pike, Bethesda, MD 20894, USA) for English language articles published from January 2000 to October 2020. MEDLINE**^®^** was queried using the following search terms (used both alone and in combination for advanced research): Imaging ocular oncology, uveal melanoma, eye irradiation, radiation chorioretinopathy, radiation retinopathy, radiation maculopathy, radiation optic neuropathy, optical coherence tomography, optical coherence tomography angiography. Additional articles were identified by reviewing the references of examined publications. To identify potentially relevant articles to include in this review, two investigators reviewed all papers, and the most significant among them were included. Articles without patients’ imaging at baseline were excluded. Review and study articles were preferred to case reports or case series. Articles included in the reference list were fully examined by the authors.

## 3. Results

### 3.1. Radiation (Chorio)Retinopathy

**Figure 4:**
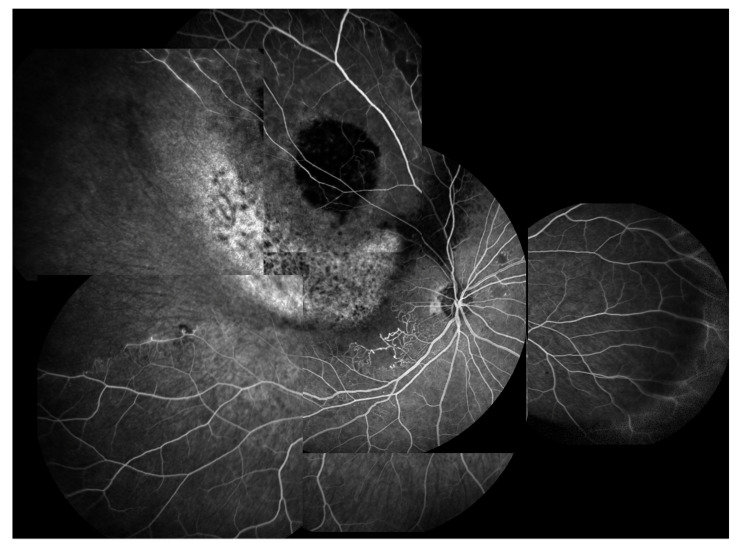
Fluorescein angiography showing chorioretinal atrophy, ischemia (capillary non-perfusion) and abnormal perifoveolar capillary network.

**Figure 5:**
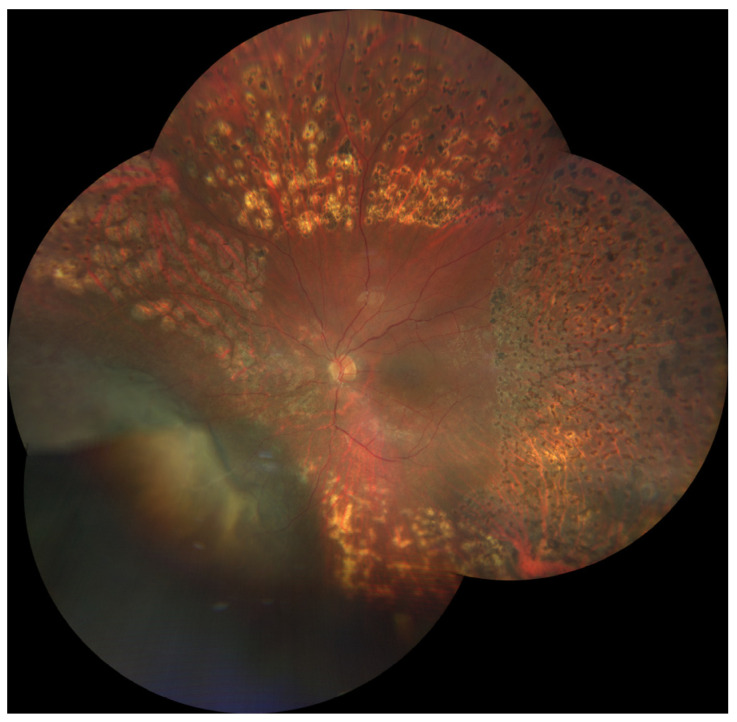
Color fundus photography of a patient treated by panretinal photocoagulation because of radiation retinopathy secondary to brachytherapy for uveal melanoma. The treated uveal melanoma is visible as a pigmented lesion (inferior nasal quadrant).

**Figure 6:**
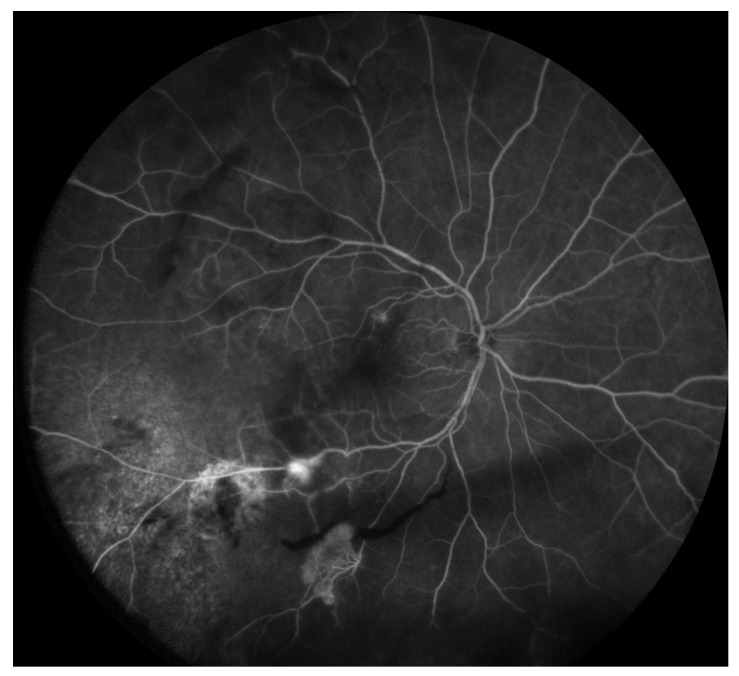
Fluorescein angiography imaging demonstrating retinal neovascularization along the inferior temporal vascular arcades bordering ischemic areas.

**Figure 7:**
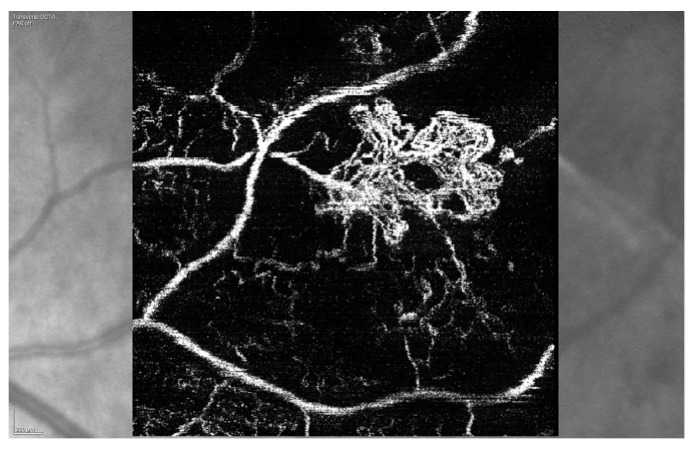
Optical coherence tomography angiography (OCTA) analysis demonstrating perifoveal neovascularization.

Radiation retinopathy (RR) (Figure 1), better defined as radiation chorioretinopathy, is one of the major complications of eye irradiation, occurring more frequently after irradiation of primary intraocular tumors. It is histopathologically characterized by progressive vascular occlusion, vascular remodeling and chorioretinal inflammation [4,5,6,7,8,9]. This pathologic process is clinically characterized by the appearance of microaneurysms and telangiectasia as well as retinal edema and hard exudates from compromised retinal capillaries. As areas of capillary dropout become confluent, retinal ischemia develops and nerve fiber layer infarctions are visible. Usually, larger vessels are involved later in the course of the disease, with marked vascular sheathing. When there are large or multiple areas of capillary non-perfusion (Figure 4), laser photocoagulation is mandatory (Figure 5) to prevent retinal and disc neovascularization (Figure 6 and Figure 7). Choroidal circulation is also directly compromised, thus suggesting that the term radiation chorioretinopathy better identifies this disorder [7].

**Figure 8:**
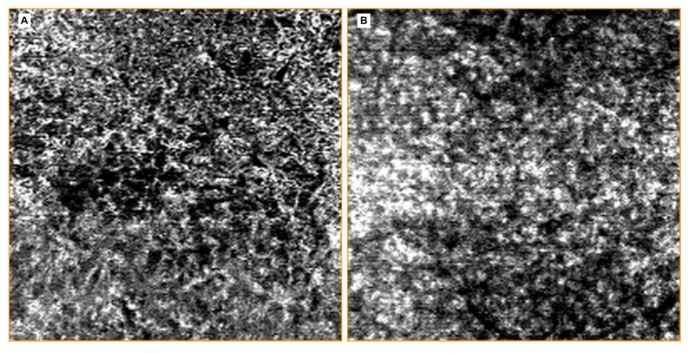
OCTA analysis of the choriocapillary vascular structure in a patient affected by radiation chorioretinopathy in his left eye (**A**). Choroidal vessels are rarefied and dilatated; “signal void” spots are recorded in the central area. The normal appearance of the choriocapillary vascular structure of the fellow-eye is showed in (**B**).

In 2005, Finger and Kurli proposed a classification of RR in four stages, using a combination of ophthalmoscopy and FA findings, offering an imaging framework to the clinical manifestations of RR [22,23]. The prominent involvement of chorioretinal vasculature, as previously described, makes RR a perfect candidate for early evaluation by OCTA [24,25]. Sellam et al. performed OCTA in irradiated eyes 36 months after the treatment for UM [24]. At the superficial capillary plexus they found: Microaneurysms (65%), capillary loss (100%) and dilated vessels (47%). Moreover, they demonstrated that the deep capillary plexus was even more impaired than the superficial one, with 100% capillary loss, 59% dilated vessels and 76% microaneurysms [24]. Shields et al. reported significant capillary dropout in superficial and deep plexuses, in addition to decreased vessel density of choriocapillaris at the tumors’ margins in patients with and without clinically evident RR [25,26]. “Signal void” spots (88%), rarefaction (94%) and dilation (41%) of the choroidal vessels were found at the level of choriocapillaris [24] (Figure 8).

In a study including 112 irradiated eyes without clinical evidence of RR, OCTA revealed a decrease in the vessel density of superficial and deep capillary plexuses, compared with the healthy fellow eyes. The authors concluded that capillary ischemia found by OCTA was the precursor of clinically evident RR [27]. Considering the previously mentioned studies, vascular abnormalities, detected using OCTA, represent the earliest changes in the clinical course of RR, with OCTA allowing a very early detection.

But, OCTA has some limitations: Large fields of view is especially challenging and alterations in blood vessels permeability, typically visualized using FA or ICGA, cannot be identified [21]. For this reason, FA remains the gold standard to detect ischemic retinal areas [16]. Finally, OCTA images may show some technical artifacts, compared to structural OCT, sometimes inducing clinical misinterpretation [21].

### 3.2. Radiation Maculopathy

Radiation maculopathy (RM) describes the specific macular involvement secondary to eye irradiation. Its relevance, from both a diagnostic and therapeutic point of view, depends on the peculiar role of the macula as visual function is concerned. Its mean onset time is 20 months after irradiation, and it may cause significant and irreversible visual loss (Figure 1 and Figure 2). Histopatologically, macular microvasculature is more vulnerable to radiation damage than all other retinal regions, because of its high capillary density. The pathologic process is clinically characterized by microaneurysms, telangiectasia, capillary non-perfusion, macular edema and hard exudates [10,18,28,29]. RM is clinically characterized by edematous changes in its early stages (cystoid macular edema) and/or by ischemia and atrophic changes in the macular area in more advanced stages.

**Figure 9:**
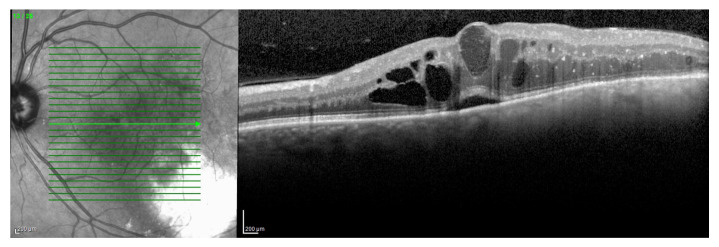
Macular OCT showing cystoid macular edema with limited subfoveal neuroretinal detachment secondary to radiation maculopathy.

Moreover, an inflammatory component in the pathophysiology of RR and RM in particular has been recently suggested, as well the presence of an “inflammatory phenotype” characterized by macular neuroretinal detachment (Figure 9) and the presence of OCT hyperreflective intraretinal foci [14].

**Figure 10:**
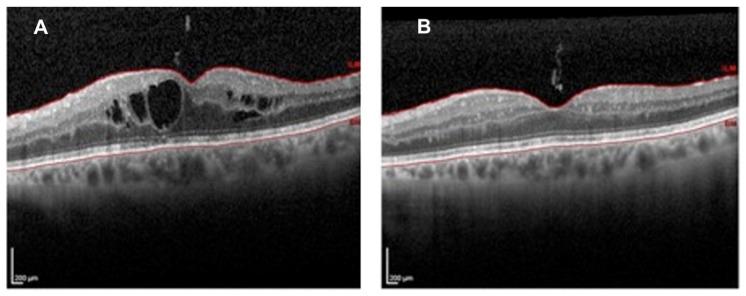
Macular OCT scans of radiation maculopathy before (**A**) and after (**B**) intravitreal treatment by dexamethasone implant: Complete resolution of macular edema was documented two months after treatment.

Horgan et al. described early morphologic changes using OCT after brachytherapy for choroidal melanoma, and found that 61% developed OCT detectable macular edema (after a mean of 12 months after irradiation), and 70% had visual acuity of 20/200 or worse [17]. More recently, Mashayekhi et al. reported subclinical macular edema (central macular thickness change >10 μm compared to the fellow eye) even before treatment in eyes with posterior UM. They demonstrated that these eyes were at greater risk for developing RM compared with eyes without the reported thickening before treatment [30]. In all reports describing OCT changes after eye irradiation for UM, OCT showed the earliest detectable signs of RM [7,17,30]. Macular edema is a key point in RM, mainly because (Figure 10) therapeutic intervention may reduce the impact on visual acuity, at least preventing irreversible visual loss [31,32].

**Figure 11:**
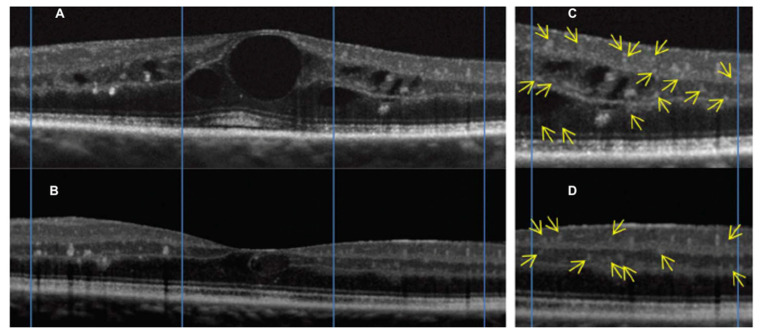
(**A**) Macular OCT linear scan in a patient affected by radiation maculopathy. (**B**) Macular OCT linear follow-up scan performed after intravitreal treatment by dexamethasone implant, showing the resolution of macular edema. (**C**) Magnification of figure A, showing hyperreflective intraretinal foci (yellow arrows). (**D**) Magnification of figure B, showing the reduction of hyperreflective intraretinal foci (yellow arrows).

Some papers have documented that massive, chronic cystoid macular edema may lead to disruption of the photoreceptor inner segment/outer segment (IS/OS) junction, one of the OCT parameters correlated with irreversible visual loss [33,34]. Thus, even with resolution of edema and restoration of near normal retinal architecture, patients often fail to regain prior visual acuity. Moreover, OCT analysis of retinal layers enables the documentation of specific reflectivity changes which have been suggested to have a relevant role in different macular diseases, such as age-related macular degeneration, edema secondary to branch vein occlusion, and diabetic retinopathy, namely: Hyperreflective intraretinal foci (Figure 11) [35]. Frizziero et al. documented, by OCT, that these hyperreflective intraretinal foci (also called spots) might be considered a novel, in vivo noninvasive biomarker to better qualify and monitor retinal inflammation in radiation-induced maculopathy [35].

**Figure 12:**
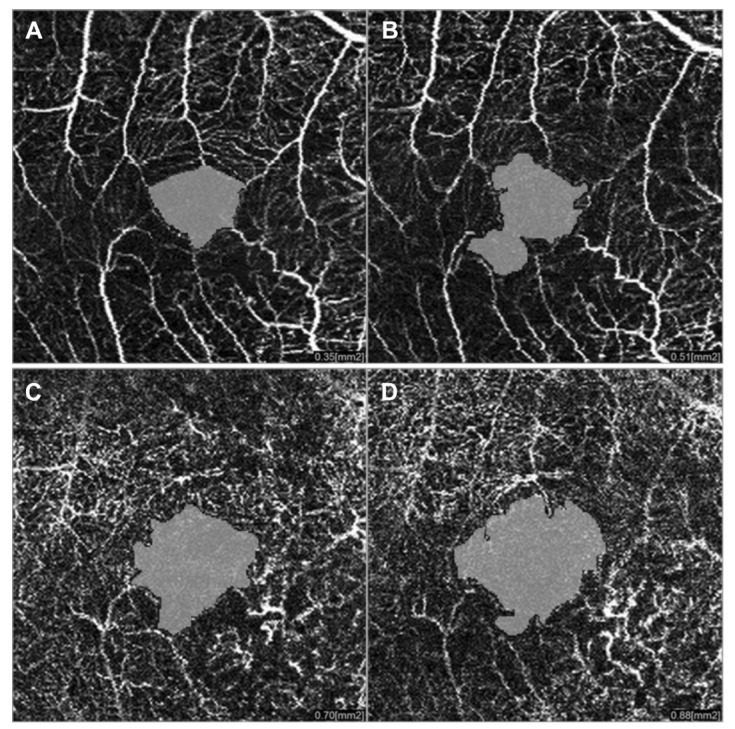
OCTA imaging of the superficial (**A**,**B**) and deep (**C**,**D**) plexuses showing foveal avascular zone enlargement in the treated eye (**A**,**C**) compared to the healthy eye (**B**,**D**) in a patient treated by brachytherapy because of posterior uveal melanoma.

**Figure 13:**
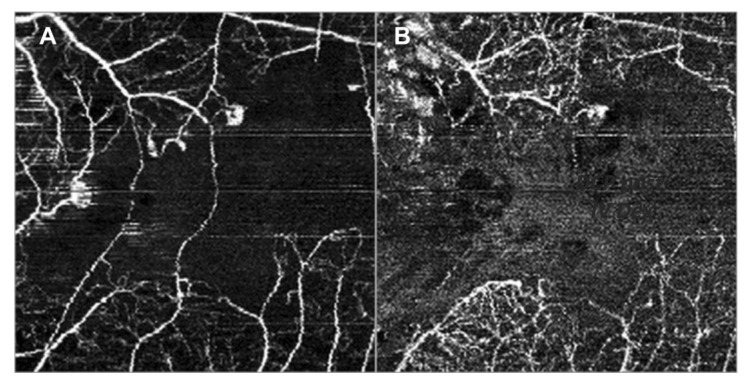
OCTA imaging of the superficial (**A**) and deep (**B**) capillary plexuses showing capillary dropout.

Consequently, OCT is currently considered the gold standard to identify and grade radiation macular edema, since its early stages. Various tentatives have been done to classify radiation maculopathy by OCT. Horgan et al. in 2010, proposed a classification of RM using Stratus OCT to grade the amount of macular edema [10]. The first limitation of this classification is the use of just a qualitative approach that may give rise to ambiguity. Furthermore, a second limitation of this study is that atrophic macular changes were not considered. Veverka et al. proposed another classification using ophthalmoscopic, OCT and OCTA findings, to consider both cystoid macular edema grading and macular ischemia [36]. They found that OCTA is able to display foveal avascular zone (FAZ) enlargement (Figure 12) and capillary dropout (Figure 13) in RM before the appearance of signs at ophthalmoscopy or OCT imaging. Unfortunately, this classification is based on just seven cases, without a formal statistical correlation with visual acuity.

Recently, our group proposed a new classification of RM using a multimodal imaging approach and a detailed multivariate statistical analysis: The cyst junction atrophy (CJA) classification. Vertical thickness of the largest maculasr cyst (C parameter), the IS/OS layer disruption (J parameter) and the presence of foveal RPE atrophy (A parameter) are the most statistically significant biomarkers related to visual acuity in patients affected by RM and can be used to clinically characterize and classify the disease stage as reported in Table 1 [37].

**Figure 14:**
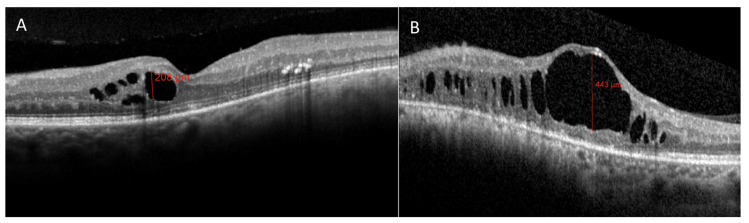
OCT imaging of patients affected by radiation maculopathy at different disease stages. (**A**) A patient affected by a relatively early-stage radiation maculopathy characterized by evident cystoid macular edema, having the vertical thickness of the largest macular cyst of 208 μm, and by the absence of the IS/OS layer disruption and RPE central atrophy (C208, J0, A0). (**B**) A patient characterized by an advanced stage radiation maculopathy characterized by evident cystoid macular edema having the vertical thickness of the largest macular cyst of 443 μm and by the presence of both the IS/OS layer disruption and RPE atrophy (C443, J1, A1).

The use of these morphologic biomarkers allows the separation of the edematous component of RM (Figure 14A), functionally relevant in relatively early disease stages from the atrophic components characterizing late stages (Figure 14B), theoretically irreversible from a functional point of view.

OCTA is very sensitive in the detection of the earliest manifestations of RM showing that very early subclinical microvascular insult occurs within the parafoveal capillary network. In 2016, Shields et al. reported their findings in 65 patients treated with Iodine-125 brachytherapy, using OCTA [25]. They demonstrated FAZ enlargement at both the superficial and deep plexuses and decreased parafoveal vessel density of both plexuses, even in eyes without clinically evident RM [25]. Similar results were reported by Sellam et al. in a small series of 17 patients [24]. Another study by Matet et al. on 93 patients demonstrated OCTA structural and microvascular changes to have significant impact on visual acuity, with worse visual acuity associated with the enlargement of the FAZ and reduced vessel density at the superficial and deep plexuses [38]. The measurement of deep vessel density may be biased by the masking effect of the overlying edema, but the results were reconfirmed after including macular thickness in the statistical analysis. Daruich et al. found that the decrease in best-corrected visual acuity and FAZ enlargement on OCTA developed in 6 months in eyes with RM, and were significantly modified by intravitreal anti-vascular endothelial growth factor treatment [39]. This preliminary study was performed on a very small number of patients and the follow-up is limited, but it highlights the usefulness of OCTA not only in the diagnosis, but also in monitoring RM. More recently, Cennamo et al. hypothesized that visual acuity irreversible changes, despite the integrity of the outer retina and the reabsorption of subretinal fluid, may be due to the absence of improvement in FAZ area and in retinal vessel density after treatment with anti-vascular endothelial growth factor [40]. The main features detectable by ophthalmoscopy, FA, OCT and OCTA are summarized in Table 2.

But, UM itself may induce metabolic stress of the retinal tissue and subsequent microischemia of the macular retinal vessels. Recent studies recorded FAZ enlargement in the deep plexus and reduced vessel density in eyes harboring UM. These vascular changes seem to be correlated with peri-tumoral subretinal fluid and large UM. Thus, the tumor itself probably stimulates the secretion of growing factors, which may lead to endothelial cell hypertrophy and capillary narrowing/closure. Moreover, the careful analysis of FAZ in the superficial plexus may help to distinguish the vascular damage due to UM from that caused by irradiation. Li et al. demonstrated that the superficial FAZ of patients with UM was unchanged compared to the fellow eye [41], while the deep FAZ was enlarged. This fact may depend on the higher vulnerability of the deep plexus (compared to the superficial one) and to the inward compression of UM that involves more the outer than the inner retina [41]. On the contrary, as already described, RM causes the enlargement of the FAZ at both the superficial and deep plexuses [41]. Probably, the loss of integrity of macular retinal vessels before irradiation may contribute to an earlier appearance of RM, but future studies are mandatory [41,42].

### 3.3. Radiation Optic Neuropathy

Radiation-induced optic neuropathy (RON) is typically characterized by a rapid visual loss occurring months to years after optic nerve irradiation [43]. The main clinical findings include: Hemorrhages and edema of the optic nerve head (Figure 1 and Figure 3). When only the post-laminar optic nerve is involved, the clinical appearance is usually subtler with gradual pallor of the optic disc [7,40]. The pathogenesis of RON includes primary radiation-induced axonal necrosis, vascular occlusion with subsequent axonal death or both [44,45]. Moreover, RON is characterized by peculiar retinal and optic disc changes that can be quantitatively evaluated by OCT, such as decreased optic cup size and increased optic disc thickness [43,46,47,48].

**Figure 15:**
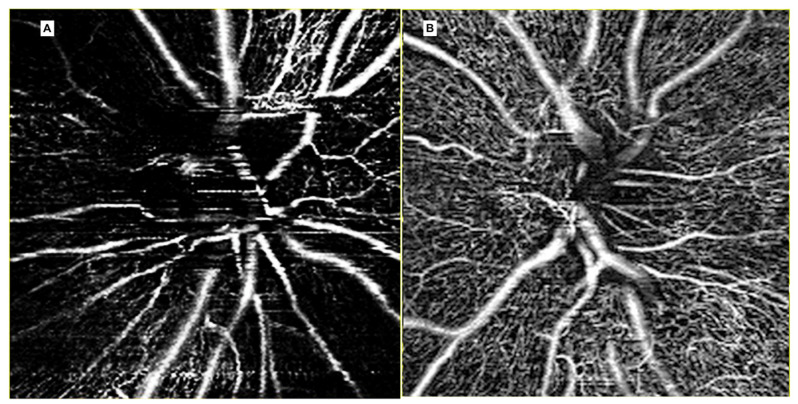
OCTA imaging of the peripapillary area. The radial peripapillary capillary plexus (RPCP) of an eye affected by radiation optic neuropathy (**A**). Note the presence of peripapillary RPCP dropout. The contralateral healthy eye is showed in (**B**).

Our group recently demonstrated that OCTA is an accurate and non-invasive method to analyze the peripapillary vascular changes secondary to RON. Both vessel area density and vessel length fraction were reduced, showing that vascular remodeling is not only characterized by a reduction of peripapillary perfusion but also by a reduction in the capillary length and number (Figure 15) [49].

Moreover, we detected radial peripapillary capillary plexus (RPCP) abnormalities to clinically classify RON, as reported in Table 3. The RPCP has a unique anatomical organization because the capillaries run parallel with the nerve fiber layer axons, as opposed to the other retinal vascular plexuses, having a lobular configuration [50,51]. Thus, the clinical detection of RPCP abnormalities is enhanced by this specific configuration. Moreover, the detection of RPCP abnormalities suggests a primary involvement of the peripapillary nerve fiber layer by RON, not only as a consequence of radiation side effects on ganglion cells.

Even Skalet et al. described the use of OCTA to evaluate RON, confirming our results: Lower peripapillary capillary density and perfusion [52]. Unfortunately, in this study RPCP and the entire peripapillary capillary bed were not analyzed separately, so RON classification was not applicable.

## 4. Discussion

Multimodal retinal imaging is a field in continuous progress. Nowadays, OCT and OCTA are the main non-invasive tools to investigate chorioretinal changes due to eye irradiation. Nevertheless, while the role of OCT for the assessment of radiation induced macular edema and optic neuropathy is quite known, the role of OCTA is still to be defined [53]. OCTA imaging, with its high-resolution and non-invasive modality, is useful for both structural and functional studies in the evaluation of chorioretinal blood flow, especially in deeper retinal and choroidal layers, which are usually masked by the overlying structures in FA. The main limitations of OCTA include its restricted field of view and the presence of possible artifacts during acquisition or post-acquisition image processing. With optimization of OCTA in visualization of peripheral retina, the suitability of this modality will surely expand not only in ocular oncology, but also in most retinal and choroidal diseases.

## 5. Conclusions

The application of OCT and OCTA technology in ocular oncology has evolved over many years by the efforts of many research groups and physicians to translate this technology from bench to bedside, and back again, with impressive results. An extraordinary application was found in OCT and OCTA for the early diagnosis and classification of radiation chorioretinopathy, maculopathy and optic neuropathy. We believe that OCTA may contribute to the early diagnosis of chorioretinal damage due to irradiation, but further research is needed.

## Figures and Tables

**Table 1:** CJA Classification of RM.

Features	Abbreviation	Grades	Grade Definition
Vertical size of the largest macular cyst *	C (cyst)	C_x_	C_x_: the vertical size of the largest macular cyst cannot be assessed; ^†^
		C_0_	C_0_: no evidence of measurable cysts;
		C_n_	C_n_: n indicate the vertical size of the largest macular cyst in μm ^‡^
IS/OS junction alterations *	J (IS/OS junction)	J_x_	J_x_: the presence of IS/OS junction alterations cannot be assessed; ^†^
		J_0_	J_0_: no evidence of IS/OS junction alterations;
		J_1_	J_1_: presence of IS/OS junction alterations;
RPE atrophy *	A (RPE atrophy)	A_x_	A_x_: the presence of RPE atrophy cannot be assessed; ^†^
		A_0_	A_0_: no evidence of RPE atrophy
		A_1_	A_1_: presence of RPE atrophy

Modified from Parrozzani et al [37]. IS/OS, photoreceptor inner segment/outer segment junction layer. * Involving the central area (1 mm). ^†^ The use of this grade should be minimized. ^‡^ For example, in a patient having the vertical size of the largest macular cysts of 534 μm, the C grade is C534.

**Table 2:** Radiation maculopathy features detectable with multimodal imaging.

Features	Ophthalmoscopy	FA	OCT	OCTA
Hemorrage	+	+/−	−	−
Microaneurysm	+	+	−	+
Telangiectasia	+	+	−	+
Hard exudate	+	+/−	+/−	−
Cotton wool spot	+	+/−	−	−
Neovasculararization	+/−	+	−	+
Hyperreflective intraretinal foci	−	−	+	−
Macular edema	+/−	+/−	+	+/−
Macular intraretinal cysts	−	+/−	+	+/−
Subfoveal fluid	−	+/−	+	−
IS/OS disruption	−	−	+	−
RPE atrophy	+/−	+/−	+	−
Vitreoretinal interface alteration	+/−	−	+	−
Macular thickness	−	−	+	−
Foveal avascular zone area	−	+/−	−	+
Macular ischemia	−	+	−	+

FA, fluorescein angiography; OCT, optical coherence tomography; OCTA, OCT angiography.

**Table 3:** Clinical classification of radiation optic neuropathy.

**Grade 0**	- Regular radial distribution of the peripapillary capillaries - Absence of vessels abnormalities
Grade 1 *	- Loss of the radial pattern of the RPCP - Absence of peripapillary ischemia
Grade 2 *	- Peripapillary hypoperfusion in less than two quadrants (defined area of RPCP dropout)
Grade 3 *	- Peripapillary hypoperfusion in more than two quadrants (RPCP dropout > 180°)
**Grade 4 ^†^**	- Peripapillary hypoperfusion, (complete RPCP dropout or poor OCT image quality)

Modified from Parrozzani et al [49]. OCT, optical coherence tomography; OCTA, OCT angiography; RPCP, radial peripapillary capillary plexus. * The evidence of interpapillomacular involvement by the detected abnormalities (for Grades 1, 2 and 3) is also defined as ‘+’ disease. ^†^ The + disease is presented by definition in Grade 4 disease.
